# The effect of vitamin D supplementation on insulin and glucose metabolism in overweight and obese individuals: systematic review with meta-analysis

**DOI:** 10.1038/srep16142

**Published:** 2015-11-06

**Authors:** Małgorzata Jamka, Małgorzata Woźniewicz, Jan Jeszka, Marcin Mardas, Paweł Bogdański, Marta Stelmach-Mardas

**Affiliations:** 1 Department of Human Nutrition and Hygiene, Poznan University of Life Sciences, 31 Wojska Polskiego Str., 60-624 Poznan, Poland; 2 Department of Oncology, Poznan University of Medical Sciences, 82/84 Szamarzewskiego Str., 60-569 Poznan, Poland; 3 Department of Education and Obesity Treatment and Metabolic Disorders, Poznan University of Medical Sciences, 84 Szamarzewskiego Str., 60-596 Poznan, Poland; 4 German Institute of Human Nutrition Potsdam-Rehbruecke, 114-116 Arthur-Scheunert-Allee, 14558 Nuthetal, Germany; 5 Department of Paediatric Gastroenterology and Metabolic Diseases, Poznan University of Medical Sciences, 27/33 Szpitalna Str., 60-572 Poznan, Poland

## Abstract

The aim of this systematic review was to assess the effect of vitamin D supplementation on glucose and insulin metabolism in overweight and obese subjects. The search process was based on the selection of publications listed in the databases: PubMed, Scopus, Web of Knowledge, Embase and the Cochrane library that met the inclusion criteria. Twelve randomized controlled trials were included. The analysed population consisted of 1181 individuals with BMIs >23 kg/m2. Changes in the concentration of 25(OH)D, fasting glucose, insulin and the HOMA-IR index were assessed. In the meta-regression analysis, a restricted maximum likelihood method was applied. To combine individual study results, a meta-analysis was performed. Vitamin D supplementation did not have an effect on glucose concentrations, insulin level and HOMA-IR values when the supplemented dose, time of supplementation and baseline of 25(OH)D concentration were taken under consideration in subgroup-analysis. This meta-analysis provides evidence that vitamin D supplementation has no significant effect on glucose and insulin metabolism in overweight and obese individuals.

According to the International Diabetes Federation (IDF), in 2013, 8.3% of adults in the world suffered from diabetes^1^. Around 80–90% of people with type 2 diabetes are obese or overweight (Body Mass Index (BMI) ≥25 kg/m2)^2^,^3^. It is well-known that obesity is related to insulin resistance and hyperinsulinemia^4^,^5^,^6^. Therefore, obesity has been recognized as one of the most important single risk factors in the pathogenesis of type 2 diabetes mellitus.

Currently, the role of vitamin D in the regulation of insulin secretion is highly investigated^7^,^8^. New findings suggest that supplementation with vitamin D could influence insulin secretion and improve glucose homeostasis^9^,^10^,^11^,^12^,^13^. Additional data suggesting that vitamin D may also have a role in delaying progression to clinical diabetes in adults at a high risk of developing type 2 diabetes, taking into consideration altered vitamin D and calcium homeostasis^13^,^14^. Vitamin D may be also necessary for proper pancreatic β-cell function which is produces the enzyme 1-α-hydroxylase involved in the conversion of 25-dihydroxyvitamin D_3_ to the active form of the hormone, 1,25-dihydroxyvitamin D_3_^15^,^16^. Although 80–100% of the required vitamin D can be provided by endogenous synthesis in the human skin, vitamin D deficiency is a common health problem and is more pronounced in the obese population^17^,^18^. Additionally, obesity is one of the most important risk factors of diabetes, gall bladder disease, hypertension, heart disease, osteoarthritis, sleep apnea, and certain forms of cancer development, which can further increase the cardiometabolic risk^19^.

The aim of this systematic review was therefore to assess the effect of vitamin D supplementation on glucose and insulin metabolism in overweight and obese subjects.

## Results

### Search results

**Figure 1:**
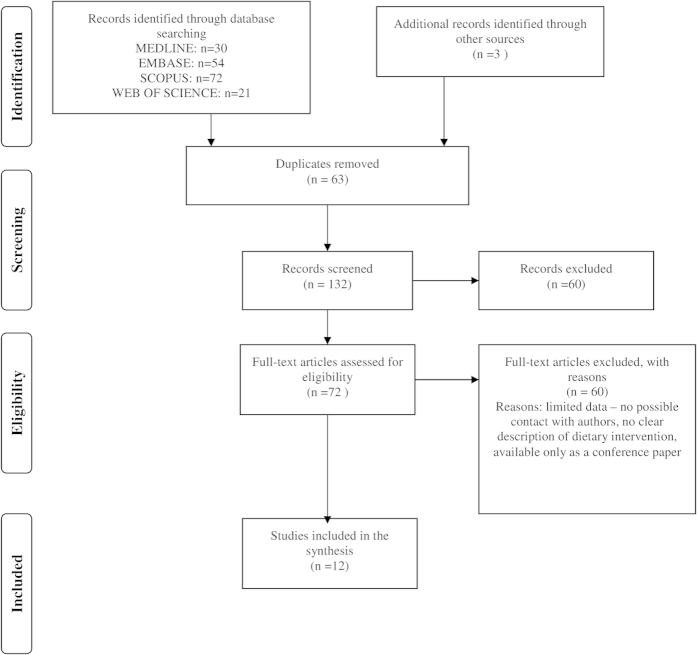
Process of the search.

Search results are presented in Fig. 1. 195 potentially relevant publications were identified; after evaluation of titles and abstracts, 69 were retrieved for full-text review. Finally, after removal of duplicates and publications with insufficient data presentation, twelve studies met the inclusion criteria and were further analysed^20^,^21^,^22^,^23^,^24^,^25^,^26^,^27^,^28^.

### Population and study characteristics

The baseline characteristics and populations of the studies are presented in Table 1 and Table 2, respectively. 1181 patients were included from the 12 selected studies. Although the date of publication of the selected papers was not constricted, all publications included in this systematic review were published after 2006. The number of individuals analysed in each study ranged from 23^24^ to 332^27^. Most of the studies were conducted in an adult population^21^,^22^,^24^,^25^,^26^,^27^,^28^,^29^ with the exception of two studies, which were performed in children and adolescents^20^,^23^. The age of subjects ranged from 9^23^ to 75^21^ years and the BMI value ranged from 23^21^ to 47^27^ kg/m2, indicating an overweight or obese population. A higher percentage of women was reported in the analysed studies; however, two studies were conducted only in men^28^,^30^ and two studies were performed only in women^24^,^29^. Considerable heterogeneity was found in the studied populations regarding ethnicity. Individuals were recruited from following ethnicities: Asian^20^,^25^,^26^,^30^, American^21^,^23^,^24^,^28^,^29^ (including African-American^21^,^23^,^28^) and European^22^,^26^,^31^. All studies were designed as randomized controlled clinical trials (RCTs). Intervention, based on supplementation with cholecalciferol, ranged from 125 IU/d^26^ to 12000 IU/d^30^ with the addition of calcium supplementation (500–1200 mg/d) in five studies^24^,^26^,^27^,^28^. The duration of supplementation varied from 12 weeks^20^,^24^,^25^,^26^,^28^ to 52 weeks^21^,^27^. Additionally, a lifestyle program, including a diet or recommendations for healthy eating and an exercise component, was incorporated in some of the studies during the intervention period^20^,^21^,^24^,^26^,^28^.

### The supplementation of vitamin D and changes in serum 25(OH**)D concentrations**

In the seven studies which assessed the effect of vitamin D supplementation on changes in 25(OH)D serum concentration, the supplemented doses of vitamin D were variable (between 1000–12000 IU/d)^20^,^21^,^22^,^23^,^24^,^25^,^26^,^27^,^28^,^29^,^30^,^31^, and in three selected studies additional calcium supplementation (500–600 mg/d) was included^20^,^21^,^24^. Baseline serum concentrations of 25(OH)D in the intervention groups ranged from 36.80 nmol/l^25^ to 54.30 nmol/l^27^ and were similar to the value observed in control groups. The indicated concentration of 25(OH)D in overweight or obese groups suggests the widespread presence of vitamin D deficiency or insufficiency in the analysed population^32^. In all individuals receiving supplementation, an increase in the serum concentration of 25(OH)D was observed, leading to a more optimal serum level^20^,^21^,^22^,^23^,^24^,^25^,^27^,^28^,^29^,^30^,^31^ (Table 3).

### Supplementation with vitamin D in relation to changes in plasma glucose concentrations, insulin levels and the Homeostatic Model Assessment of Insulin Resistance (HOMA-IR) index

**Figure 2:**
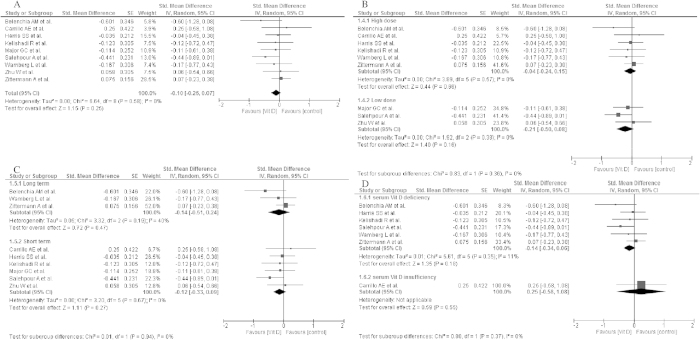
Forest plot of the random-effects meta-analysis of changes in glucose concentration according to (A). Overall effect (**B**). The dose of vitamin D supplementation (**C**). Time of vitamin D supplementation and (**D**). Baseline of 25(OH)D concentration shown as polled standard differences in the means with 95% CI by standard differences in means of glucose concentrations in selected randomised trials. *For each study, the square represents the point estimate of the intervention effect. Horizontal lines join the lower and upper limits of the 95% CI of this effect. The area of shaded squares reflects the relative weight of the study in the meta-analysis. Diamonds represent the subgroup mean difference and pooled mean differences. CI indicates confidence interval. Low dose: 125–2000 IU/day; high dose: 3571–4000 IU/d, deficiency: <50 nmol/l, insufficiency:52.5–72.5 nmol/l, short: until 15-weeks, long: >15weeks.

Changes in fasting plasma glucose concentrations after vitamin D supplementation were analysed in nine studies deemed eligible during the search process^20^,^23^,^24^,^25^,^26^,^28^,^29^,^30^,^31^. Average baseline blood glucose concentration ranged from 4.55 mmol/l^26^ to 5.67 mmol/l^31^, in range with Normal Fasting Glucose (NFG) or indicated Impaired Fasting Glucose (IFG)^32^ values. Following the intervention period, mean glucose concentrations decreased in individuals who had received vitamin D supplementation in seven selected studies^20^,^23^,^24^,^25^,^28^,^30^,^31^, while two studies showed no differences in blood glucose levels^26^,^30^ (Table 4). However, vitamin D supplementation failed to show a significant effect on glucose concentrations (std differences: −0.10, 95% CI: −0.26, 0.07, p = 0.25) overall, as well as in the subgroups meta-analysis taking into consideration: the dose of vitamin D supplementation (high dose: std differences: −0.04; 95% CI: −0.24, 0.15; p = 0.66; low dose: std differences: −0.21; 95% CI: −0.50, 0.08; p = 0.16), time of vitamin D supplementation (long term: std differences: −0.14; 95% CI: −0.51, 0.24; p = 0.47; short term: std differences: −0.12; 95% CI: −0.33, 0.09; p = 0.27) and baseline 25(OH)D concentration (deficiency: std differences: −0.14; 95% CI: −0.34, 0.06; p = 0.18; insufficiency: std differences: 0.25; 95% CI: −0.58, 1.08; p = 0.55) (Fig. 2).

**Figure 3:**
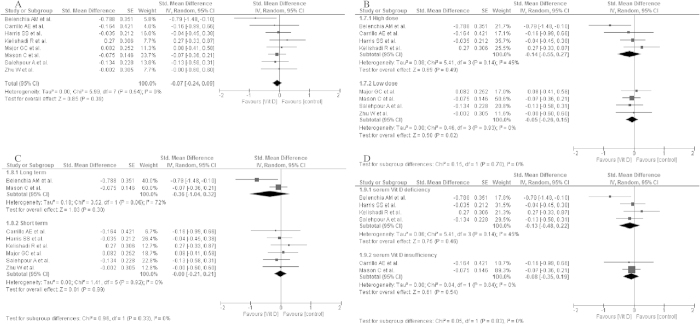
Forest plot of the random-effects meta-analysis of changes in insulin concentration according to (A). Overall effect (**B**). The dose of vitamin D supplementation (**C**). Time of vitamin D supplementation and (**D**). Baseline of 25(OH)D concentration shown as polled standard differences in means with 95% CI by standard differences in means of glucose concentrations in selected randomised trials. *For each study, the square represents the point estimate of the intervention effect. Horizontal lines join the lower and upper limits of the 95% CI of this effect. The area of shaded squares reflects the relative weight of the study in the meta-analysis. Diamonds represent the subgroup mean difference and pooled mean differences. CI indicates confidence interval. Low dose: 125–2000 IU/day; high dose: 3571–4000 IU/d, deficiency: <50 nmol/l, insufficiency:52.5–72.5 nmol/l, short: until 15-weeks, long: >15weeks.

The influence of vitamin D supplementation on insulin secretion was evaluated in nine studies included in this systematic review^20^,^21^,^22^,^23^,^24^,^25^,^27^,^28^,^30^. In groups receiving vitamin D supplementation (D groups), the mean fasting plasma insulin concentrations ranged from 56.30 pmol/l^24^ to 160.40 pmol/l^22^. After the intervention, the mean fasting insulin levels decreased in individuals from seven studies in the D groups^20^,^21^,^22^,^24^,^25^,^28^,^30^; however, in two studies, an increase was observed^22^,^26^. A similar trend was indicated in the control groups (Table 4). In the subgroup meta-analysis, no significant effect of vitamin D supplementation on insulin levels was shown (std differences: −0.07, 95% CI: −0.24, 0.09, p = 0.39) as well as when taking into consideration: the dose of vitamin D supplementation (high dose: std differences: −0.14; 95% CI: −0.55, 0.27; p = 0.49; low dose: std differences: −0.05; 95% CI: −0.26, 0.15; p = 0.62), time of vitamin D supplementation (long term: std differences: −0.36; 95% CI: −1.04, 0.32; p = 0.30; short term: std differences: 0.00; 95% CI: −0.21, 0.21; p = 0.92) and baseline of 25(OH)D concentration (deficiency: std differences: −0.13; 95% CI: −0.48, 0.22; p = 0.46; insufficiency: std differences: −0.08; 95% CI: −0.35, 0.19; p = 0.54) (Fig. 3).

**Figure 4:**
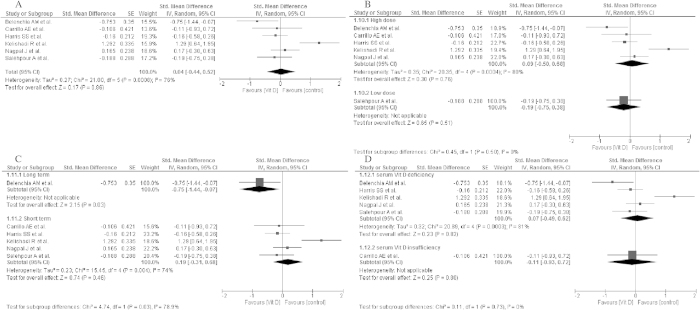
Forest plot of the random-effects meta-analysis of changes in HOMA-IR according to (A). Overall effect (**B**). The dose of vitamin D supplementation (**C**). Time of vitamin D supplementation and (**D**). Baseline of 25(OH)D concentration shown as polled standard differences in means with 95% CI by standard differences in means of glucose concentrations in selected randomised trials. *For each study, the square represents the point estimate of the intervention effect. Horizontal lines join the lower and upper limits of the 95% CI of this effect. The area of shaded squares reflects the relative weight of the study in the meta-analysis. Diamonds represent the subgroup mean difference and pooled mean differences. CI indicates confidence interval. Low dose: 125–2000 IU/day; high dose: 3571–4000 IU/d, deficiency : <50 nmol/l, insufficiency:52.5–72.5 nmol/l, short: until 15-weeks, long: >15weeks.

The effects of vitamin D supplementation on the HOMA-IR index were analysed in eight selected studies^20^,^22^,^23^,^24^,^25^,^27^,^28^. At baseline in all individuals from the included studies, the mean values of the HOMA-IR index exceeded a value of 1.8, indicating insulin resistance^33^. In most patients, the values of the HOMA-IR index decreased after vitamin D supplementation^20^,^22^,^23^,^25^,^27^,^29^, with the exception of two studies^24^,^28^. Changes in the HOMA-IR index in control groups were variable^24^,^28^(Table 4). Due to incomplete data in the meta-analysis, the results from seven studies were analysed, which did not show any significant effect of vitamin D supplementation on the HOMA-IR index (std differences: 0.04; 95% CI: −0.44, 0.52; p = 0.86) as well as when taking into consideration the results from subgroups analysis: the dose of vitamin D supplementation (high dose: std differences: 0.09; 95% CI: −0.50, 0.69; p = 0.76; low dose: std differences: −0.19; 95% CI: −0.75, 0.38; p = 0.51), time of vitamin D supplementation (long term: std differences: −0.75; 95% CI: −1.44, −0.07; p = 0.03; short term: std differences: 0.19; 95% CI: −0.31, 0.68; p = 0.46) and baseline 25(OH)D concentration (deficiency: std differences: 0.07; 95% CI: −0.49, 0.62; p = 0.82; insufficiency: std differences: −0.11; 95% CI: −0.93, 0.72; p = 0.80) (Fig. 4).

**Figure 5:**
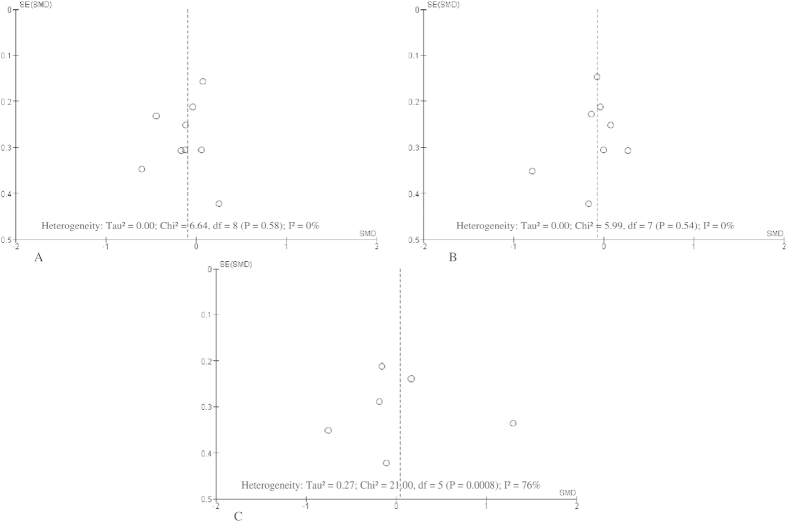
Funnel plot of standard error by standard differences in means of plasma (A). Glucose concentrations (**B**). Plasma insulin concentrations (**C**). HOMA-IR in selected randomised trials. *The summary diamonds at the bottom of the plot represent the summarized effects using fixed and random effects models, where the random effects estimates are considered the primary findings for this study due to heterogeneity.

The effects were plotted against their standard error in the funnel plots (Fig. 5).

### Meta-regression analysis of the dose-response effect

Examination of whether the impact of daily supplementation with vitamin D could predict differences between groups (Figures 6–8 in supplemental file) showed no significant association (p > 0.05).

There was no significant association between A. supplemented dose of vitamin D (β = 0.000; SE: 0.000, 95% CI:−0.0001−0.0001; z = 0.31; p = 0.7562) B. time of vitamin D supplementation (β = 0.0049; SE: 0.0048, 95% CI:−0.0045- 0.0144; z = 1.02; p = 0.3081) C. baseline of 25(OH)D concentration (β = −0.0101; SE: 0.0147, 95% CI:−0.0389 – 0.0187; z = −0.69; p = 0.4915) and mean differences in glucose levels (Figure 6-supplemental file).

Similarly, there was no significant association between A. supplemented dose of vitamin D (β = −0.000; SE: 0.0001, 95% CI:−0.0002−0.0001; z = −0.58; p = 0.5644) B. time of vitamin D supplementation (β = −0.0017; SE: 0.0046, 95% CI:−0.0108− 0.0074; z = −0.36; p = 0.7175) C. baseline of 25(OH)D concentration (β = −0.0017; SE: 0.0138, 95% CI:−0.0286 – 0.0253; z = −0.12; p = 0.9039) and insulin level (Figure 7-supplemental file).

No significant association between A. supplemented dose of vitamin D (β = 0.000; SE: 0.0001, 95% CI:−0.0001−0.0002; z = 0.20; p = 0.8386) B. time of vitamin D supplementation (β = −0.0518; SE: 0.0427, 95% CI:−0.135- 0.032; z = −1.21; p = 0.2253) C. baseline of 25(OH)D concentration. β = −0.0013; SE: 0.0522, 95% CI:−0.103 – 0.101; z = −0.33; p = 0.9794) and the HOAM-IR index was found (Figure 8-supplemental file).

## Discussion

The findings of the systematic review presented here show no statistically significant impact of vitamin D supplementation on glucose and insulin levels or the HOMA-IR index taking into consideration dose, time of vitamin D supplementation and baseline concentration of vitamin D in serum. This is one of the first reviews summarizing randomized controlled trials that investigated the influence of vitamin D supplementation on glucose and insulin metabolism in overweight and obese populations.

In our analysis, the studied population was characterized as overweight or obese, which was related to low baseline serum concentrations of 25(OH)D, indicating vitamin D deficiency or insufficiency^32^. Similar results were previously confirmed in individual studies^8^,^34^,^35^. Vimaleswaran *et al.*^35^ showed that each unit (kg/m2) increase in BMI was associated with a 1.15% lower concentrations of 25(OH)D. Nevertheless, within the selected RCTs, we did not find any information on long-term changes in 25(OH)D concentrations in obese individuals that could significantly support the merit of dietary intervention in the treatment of vitamin D deficiency. The inconsistency of results from the analysed studies could be due to the variable doses of vitamin D (up to 12000 IU/day^30^) and additional calcium supplementation. Additionally, in three selected studies^20^,^27^,^30^, vitamin D was administered once weekly, but according to the Endocrine Society, vitamin D given either once a day or once a week is effective in the achievement a level of 25(OH)D above 50 nmol/l^32^. However, improving the nutritional status of vitamin D did not translate into an improvement in glucose and insulin metabolism. The facts are consistent with data from the recently published meta-analysis by Seida *et al.*^36^ where no effect of vitamin D3 supplementation on glucose homeostasis or diabetes prevention was shown. Further, Gagnon *et al.*^37^ confirmed that although daily vitamin D and calcium supplementation may not change insulin secretion and β-cell function in adults at risk of type 2 diabetes, it may improve insulin sensitivity in patients with prediabetes. Moreover, Wood *et al*^34^ indicated that even improving vitamin D status through dietary supplementation is unlikely to reduce cardiovascular diseases risk factors.

It has previously been shown in that body weight reduction leads to an improvement in biomarker concentrations (e.g. cholesterol, glucose and insulin)^35^,^38^. Therefore, some authors have suggested that the observed positive results in single studies were most likely a consequence of energy restriction and changes in body weight in the studied individuals^28^. In the random-effects analysis of changes either in glucose or insulin according to the dose of vitamin D supplementation, we were not able to show statistically significant effects. There is still some conjecture as to whether calcium and vitamin D, alone or in combination, can affect insulin-secreting cells. It is known that insulin secretion is a calcium-dependent process; thus, vitamin D may influence pancreatic β-cells via the regulation of calcium concentrations^39^.

When interpreting the results, one should also take into consideration the number of studied populations, which does not always give sufficient power to the statistical tests and therefore clear conclusions about a negative or positive association between the analysed variables cannot be made. The results might also vary depending on the duration of the intervention period. The optimal time of intervention that is necessary to evaluate the effects of vitamin D supplementation on parameters related to glucose and insulin metabolism is not well established. However, in individuals with diagnosed vitamin D deficiency, the most common recommended duration of supplementation is eight weeks. During this period, the blood concentration of 25(OH)D should exceed the recommended value 72.5 nmol/l^32^. In selected studies, the beneficial influence of supplementation was visible after 12–52 weeks of intervention^20^,^21^,^22^,^23^,^24^,^25^,^26^,^27^,^28^,^29^,^30^,^31^. This discrepancy could be the main reason that the analysed data in the random-effects meta-analysis of changes in the HOMA-IR index according to the dose of vitamin D supplementation did not show any statistically significant effect. Of note, the mean values of the HOMA-IR index, both before and after the intervention period, indicated a high incidence of insulin resistance in the analysed population, which is highly prevalent in overweight and obese individuals^4^,^5^,^6^. It should be highlighted that these results could also be affected by other factors such as age, season and ethnicity^17^,^40^,^41^. It might be meaningful to supply higher doses of vitamin D from exogenous sources in adult and elderly people in order to obtain similar plasma levels as in younger individuals^32^. Similarly, a higher production of cholecalciferol is found in the summer months than in the winter months^42^, and in Caucasian populations compared to others^41^. The analysed studies were performed in different ethnic populations, and the season in which they were conducted was not often reported. Therefore, in this systematic review, considerable heterogeneity of analysed results was present. Meta-regressions provide a convenient way to jointly assess the effects of several factors, when an appropriate model is used.

Finally, we have to consider whether vitamin D supplementation can really be an effective treatment for the improvement of glucose metabolism. Data from a cohort study published by Sollid *et al.*^43^ did not improve glycemic indices, blood pressure, or lipid status in subjects with impaired fasting glucose and/or impaired glucose tolerance after 1-year of vitamin D supplementation at high doses (20,000IU/week). Further, an effect of vitamin D supplementation on glucose metabolism was not indicated in some studies performed in patients with type 2 diabetes^42^,^44^. Although the change in glucose metabolism in obese or pre-diabetic patients should be easy detected, the results are also uncertain^23^,^28^.

Several limitations should be listed regarding this systematic review. Firstly, it could be that some studies published in the grey literature were omitted in the literature search. Secondly, the number of individuals who participated in most of the included studies were relatively small. Additionally, combined supplementation of vitamin D with calcium makes it difficult to distinguish the effects of those two components. Furthermore, the doses of vitamin D supplied in the analysed studies, as well as the duration of supplementation, were different and could influence the full interpretation of the collected data.

In conclusion, this systematic review provides an evidence that supplementation with vitamin D has no significant effect on glucose and insulin metabolism in overweight and obese individuals but positively influences the serum concentration of 25(OH)D.

## Methods

### Search strategy

In the period from December 2014 to January 2015, the following databases were systematically searched: PubMed (Medline), Scopus, Web of Knowledge, Embase and the Cochrane library. Through the search process, we identified publications describing the effect of vitamin D supplementation on glucose or insulin metabolism in overweight and obese individuals. The search was restricted to the human population, in the English language and intervention studies, including both randomized and non-randomized controlled trials. Only reviews and original articles were eligible for this review. The date of study publication was not limited. There was also no restriction based on the age of individuals. The search was based on the following index terms and titles or abstracts: *“vitamin D OR ergocalciferol OR cholecalciferol OR 25-hydroxyvitamin D 2 OR hydroxycholecalciferol OR calcifediol OR calcitriol OR 24,25-dihydroxyvitamin D 3 OR dihydrotachysterol” AND “dietary supplements” AND “obesity OR obesity, morbid OR obesity, abdominal OR overweight” AND “insulin OR insulin resistance OR insulin-secreting cells OR blood glucose” NOT “animals”.*

The systematic review protocol was registered in the PROSPERO International prospective register of systematic reviews with the registration number CRD42014015366^45^. The PRISMA Statement was followed^46^.

### Inclusion and exclusion criteria

Inclusion criteria were as follows: studies conducted in overweight or obese individuals with type 2 diabetes, prediabetes (IFG, impaired glucose tolerance (IGT)), and normal glucose tolerance (NGT) or with NFG.

For the exclusion criteria, the following were considered: studies conducted in pregnant or breast-feeding women, in individuals suffering from type 1 diabetes, hepatic disease or kidney disease or with a history of bariatric surgery, studies conducted in animal models, articles available only in abstract form (no possible contact with authors) and containing no original research, observational studies (cohort study, case-control study, case reports, case series), studies in any language other than English, studies not documenting an association between vitamin D supplementation and glucose homeostasis, insulin secretion or insulin resistance, studies performed in subjects with a normal body weight (BMI < 25.0 kg/m2 or < 23.0 kg/m2 for Asian populations)^47^,^48^.

### Data extraction and analysis

Each of the databases was searched in parallel by two independent researchers based upon the inclusion and exclusion criteria. Review and original articles were assessed according to the title, abstract and full text in subsequent stages. In the next step, studies deemed useful by at least one of reviewers were incorporated. Doubts were resolved by review team by consensus^40^. Each selected publication was studied critically. For a quality assessment of questionable articles, the checklist described by Kmet *et al.*^33^ was used.

In the assessment of the changes in serum 25(OH)D concentrations after vitamin D supplementation, the recommendations proposed by Endocrine Society^32^ were used as they can be applied to the general population and groups at risk of vitamin D deficiency. Vitamin D deficiency was defined as serum 25(OH)D concentrations <50 nmol/l, insufficiency: 52.5–72.5 nmol/l, and sufficiency ≥72.5 nmol/l^32^.

The recommendations of the American Diabetes Association (ADA) were used to assess fasting plasma glucose and insulin concentrations. IGT is defined as a plasma concentration of glucose at 120 minutes in the oral glucose tolerance test (OGTT) ranging from 7.8 to 11.0 mmol/l, IFG is defined as a fasting plasma glucose concentration from 5.6 to 6.9 mmol/l, NGT is defined as a plasma level of glucose at 120 minutes in the OGTT < 7.8 mmol/l and NFG is defined as a fasting plasma glucose level ranging from 3.9 to 5.5 mmol/l^49^. A reference range for fasting insulin of <174 pmol/l was assumed^50^.

The changes in the HOMA-IR index during supplementation were used to assess the alterations in insulin resistance within the studied populations. According to ATP III-Met we defined cut-off values of HOMA-IR for the diagnosis of insulin resistance as ≥1.8^51^.

### Statistical analysis

To combine individual study results, a meta-analysis was performed. Data were analysed using a random-effects model, which allowed that the true effect could vary from study to study. The effect sizes in the studies that actually were performed are assumed to represent a random sample of these effect sizes. The effect size was investigated using standardized mean difference with a 95% confidence interval. The standardized mean difference transforms all effect sizes from each study to a common metric. We applied a restricted maximum likelihood (REML) based meta-regression analysis to check whether the dose of vitamin D supplementation could predict changes in glucose concentrations, insulin levels and the HOMA-IR index. The analysis was performed using Comprehensive Meta-Analysis software (Biostat, Inc., Engelwood, US) by applying an REML method to estimate the between-study variance^52^. This method corresponds to random-effects meta-regression including both within-study variances of treatment effects and the residual between-study heterogeneity. The results of the meta-analysis were visualized using a forest plot which illustrates the results of the individual studies and the summary effect.

## Additional Information

**How to cite this article**: Jamka, M. *et al.* The effect of vitamin D supplementation on insulin and glucose metabolism in overweight and obese individuals: systematic review with meta-analysis. *Sci. Rep.* **5**, 16142; doi: 10.1038/srep16142 (2015).

## Supplementary Material

Supplementary File

## Figures and Tables

**Table 1:** Characteristics of the included studies.

Study	Year	Country	Study design	Subjects (n)^a^	Intervention	Supplemented dose of vitamin D (IU/d)	Time of intervention
Kelishadi R. *et al.*^20^	2014	Iran	RCT^b^	43	Cholecalciferol^c^	3571^f^	12 weeks
Mason C. *et al.*^21^	2014	USA	RCT^b^	188	Cholecalciferol weight loss intervention^d^	2000	52 weeks
Wamberg L. *et al.*^22^	2013	Denmark	RCT^b^	52	Cholecalciferol	7000	26 weeks
Belenchia A. M. *et al.*^23^	2013	USA	RCT^b^	35	Cholecalciferol	4000	26 weeks
Carrillo A. E. *et al.*^24^	2013	USA	RCT^b^	23	Cholecalciferol calcium resistance training^e^	4000^g^	12 weeks
Salehpour A. *et al.*^25^	2013	Iran	RCT^b^	77	Cholecalciferol	1000	12 weeks
Zhu W. *et al.*^26^	2013	China	RCT^b^	43	Cholecalciferol calcium energy restriction diet (−500 kcal/d)	125^g^	12 weeks
Beilfuss J. *et al.*^27^	2012	Norway	RCT^b^	332	Cholecalciferol calcium	2857/5714^f^,^g^	52 weeks
Harris S. S. *et al.*^28^	2012	USA	RCT^b^	89	Cholecalciferol calcium	4000^g^	12 weeks
Zittermann A. *et al.*^31^	2009	Germany	RCT^b^	165	Cholecalciferol^c^	3332	52 weeks
Nagpal J. *et al.*^30^	2009	India	RCT^b^	71	Cholecalciferol	12000	6 weeks
Major G. C. *et al.*^29^	2007	Canada	RCT^b^	63	Cholecalciferol calcium energy restriction diet (−700 kcal/d)	400^g^	15 weeks

^a^Number of subjects who completed the study.

^b^Randomized Controlled Trial.

^c^Both groups also received recommendations for healthy eating and reduction of sedentary activities.

^d^Lifestyle program included a diet (total daily energy intake of 1200 to 2000 kcal/d based on baseline weight, 30% daily energy intake from fat) and an exercise component (45 min of moderate-to-vigorous intensity exercise 5 d/wk (225 min/wk)).

^e^Resistance training included: treadmill exercises and eight resistance exercises (leg extension, leg flexion, leg press, hip adduction, hip abduction, chest press, seated row, and lateral pull down).

^f^Vitamin D was administered once a week.

^g^Additional calcium supplementation (500-1200 mg/d).

**Table 2:** Characteristics of the study populations (n = 1181).

Study	Subjects (n)^a^	Analysed groups	Age (years) Mean ± SD	BMI (kg/m^2^) Mean ± SD	Sex (% of women)	Race/Ethnicity
Kelishadi R. *et al.*^20^	43	D group^b^ Control group	N/A	28.1 ± 1.1 27.8 ± 1.0	52	Asian-100%
Mason C. *et al.*^21^	188	D group^b^ Control group	60.3 ± 5.3 59.0 ± 4.7	32.3 ± 5.5 32.5 ± 6.1	93	Non-Hispanic white-86% Non-Hispanic black-6%, Hispanic-2% Other-6%
Wamberg L. *et al.*^22^	52	D group^b^ Control group	39.5 ± 8.0 41.2 ± 6.8	36.1 ± 3.4 35.0 ± 3.2	71.1	European-100%
Belenchia A. M. *et al.*^23^	35	D group^b^ Control group	14.6 ± 2.3 13.9 ± 2.4	39.5 ± 5.1 38.9 ± 6.7	50	African American-30%
Carrillo A. E. *et al.*^24^	23	D group^b^ Control group	26.2 ± 5.1 26.0 ± 4.5	30.6 ± 3.1 31.9 ± 3.3	100	American-100%
Salehpour A. *et al.*^25^	77	D group^b^ Control group	38.0 ± 7.0 37.0 ± 8.0	30.1 ± 3.9 29.5 ± 4.4	50	Asian-100%
Zhu W. *et al.*^26^	43	D group^b^ Control group	20.1 ± 1.1 20.3 ± 0.8	26.0 ± 1.8 25.7 ± 1.7	61	Asian-100%
Beilfuss J. *et al.*^27^	332	D group^b^ Control group	50.0	33.5 34.7	43 56	European-100%
Harris S. S. *et al.*^28^	89	D group^b^ Control group	57.0 ± 10.4 56.3 ± 12.3	32.6 ± 4.1 31.9 ± 4.0	0	African American-100%
Zittermann A. *et al.*^31^	165	D group^b^ Control group	47.4 ± 10.3 48.8 ± 10.1	33.7 ± 4.1 33.0 ± 4.3	N/A	European-100%
Nagpal J. *et al.*^30^	71	D group^b^ Control group	42.4 ± 6.6 45.0 ± 9.2	26.7 ± 4.54 26.0 ± 3.46	0	Indian-100%
Major G. C. *et al.*^29^	63	D group^b^ Control group	43.6 ± 5.0 41.6 ± 6.1	31.4 ± 2.5 32.3 ± 3.54	100	American-100%

^a^Number of subjects who completed the study.

^b^Group receiving vitamin D supplementation, N/A - not available.

**Table 3:** Mean changes in serum vitamin D concentration during supplementation with vitamin D in the intervention and control groups in selected studies.

Study	Supplemented dose of vitamin D (IU/d)	Analysed groups	Serum 25(OH)D (nmol/l) concentration Mean ± SD	
			Baseline	Intervention
Salehpour A. *et al.*^25^	1000	D group^b^ Control group	36.80 ± 30.00 46.90 ± 32.00	75.00 ± 22.00 51.50 ± 31.00
Mason C. *et al.*^21^	2000	D group^b^ Control group	53.40 ± 15.50 53.40 ± 15.20	87.40 ± 23.50 50.20 ± 16.70
Beilfuss J. *et al.*^27^	2857/5714c,^d^	D group^b^ Control group	54.30 (15.40–111.50)^a^ 52.40 (18.50–99.40)^a^	99.00 (46.70–193.40)^a^ 50.00 (20.30–99.80)^a^
Zittermann A. *et al.*^31^	3332	D group^b^ Control group	30.0 ± 17.5 30.3 ± 20.1	85.5 ± 57.5 42.0 ± 35.0
Kelishadi R. *et al.*^20^	3571^c^	D group^b^ Control group	45.60 ± 5.09 44.70 ± 5.66	79.89 ± 5.34 47.59 ± 5.02
Belenchia A. M. *et al.*^22^	4000	D group^b^ Control group	47.90 ± 15.70 48.90 ± 19.70	99.80 54.90
Carrillo A. E. *et al.*^24^	4000^d^	D group^b^ Control group	51.90 ± 20.70 45.20 ± 16.20	83.40 ± 18.00 58.70 ± 15.00
Harris S. S. *et al.*^28^	4000^d^	D group^b^ Control group	39.60 ± 12.90 38.20 ± 15.50	81.10 ± 27.90 37.40 ± 16.10
Wamberg L. *et al.*^22^	7000	D group^b^ Control group	33.0 ± 10.8 34.0 ± 9.0	110.2 ± 21.2 46.8 ± 17.3
Nagpal J. *et al.*^30^	12000	D group^b^ Control group	35.1 ± 27.28^e^ 0.65 ± 11.78	

*The changes in the serum 25(OH)D concentration between baseline and the end of intervention were statistically significant (p < 0.05) in the D groups of each analysed study.

^a^Mean and range.

^b^Group receiving vitamin D supplementation.

^c^Vitamin D was administered once a week.

^d^Additional calcium supplementation (500–600 mg/d).

^e^Changes where p < 0.0001.

**Table 4:** Mean changes in plasma glucose concentration, insulin level and value of the HOMA-IR index during supplementation with vitamin D in the intervention and control groups in selected studies.

Study	Supplemented dose of vitamin D (IU/d)	Analysed groups	Fasting glucose (mmol/l) Mean ± SD		Fasting insulin (pmol/l) Mean ± SD		HOMA-IR Mean ± SD	
			Baseline	Intervention	Baseline	Intervention	Baseline	Intervention
Zhu W. *et al.*^26^	125^d^	D group^b^	4.62 ± 0.26	4.89 ± 0.37^*^	72.85 ± 35.00	64.17 ± 37.22	N/A	N/A
		Control group	4.55 ± 0.34	4.91 ± 0.31	62.99 ± 27.92	64.10 ± 32.08		
Major G. C. *et al.*^29^	400^d^	D group^b^	5.66 ± 0.44	5.53 ± 0.38*	114.00 ± 44.50	100.10 ± 42.80*	N/A	N/A
		Control group	5.60 ± 0.37	5.49 ± 0.31	114.80 ± 54.60	103.90 ± 47.50		
Salehpour A. *et al.*^25^	1000	D group^b^	4.70 ± 0.50	4.40 ± 0.50*	56.30 ± 22.40	31.40 ± 18.90	12.10 ± 5.50	6.30 ± 4.10
		Control group	4.90 ± 0.40	4.20 ± 0.40	48.10 ± 25.60	29.10 ± 15.20	10.60 ± 6.20	5.60 ± 3.30
Mason C. *et al.*^21^	2000	D group^b^	N/A	N/A	75.42 (68.75, 82.64)^f^	58.20 (52.09, 64.59)^f^^*^	N/A	N/A
		Control group			76.81 (69.45, 85.42)^f^	60.14 (53.48, 67.37)^f^		
Beilfuss J. *et al.*^27^	2857/5714c,^d^	D group^b^	N/A	N/A	N/A	N/A	3.74 (0.8–17.4)^a^	3.48 (0.54–42.98)^a^
		Control group					4.10 (1.19–16.76)^a^	4.12 (1.16–31.38)^a^
Zittermann A. *et al.*^31^	3332	D group^b^	5.67 ± 0.78	5.44 ± 0.61	N/A	N/A	N/A	N/A
		Control group	5.67 ± 1.17	5.39 ± 0.72				
Kelishadi R. *et al.*^20^	3571^c^	D group^b^	5.24 ± 0.30	5.04 ± 0.25*	99.10 ± 9.17	95.21 ± 10.97*	3.21 ± 0.11	2.81 ± 0.25*
		Control group	5.12 ± 0.35	5.00 ± 0.31	98.54 ± 8.33	97.72 ± 7.22	3.15 ± 0.26	3.07 ± 0.14
Belenchia A. M. *et al.*^23^	4000	D group	5.00 ± 0.10	4.70 ± 0.10*	160.40 ± 11.80	115.30 ± 13.90*	5.12 ± 0.40	3.49 ± 0.46*
		Control group	4.90 ± 0.10	4.90 ± 0.10	150.00 ± 12.50	158.30 ± 13.20	4.79 ± 0.43	5.05 ± 0.46
Carrillo A. E. *et al.*^24^	4000^d^	D group^b^	5.40 ± 0.50	5.10 ± 0.40	108.50 ± 62.30	124.30 ± 69.50	3.80 ± 2.30	4.10 ± 2.30
		Control group	5.30 ± 0.40	5.20 ± 0.40	105.80 ± 71.20	114.90 ± 46.20	3.60 ± 2.40	3.90 ± 1.50
Harris S. S. *et al.*^28^	4000^d^	D group^b^	5.23 ± 0.44	5.20	90.20 ± 38.80	97.90	3.48 ± 1.51	3.80
		Control group	5.47 ± 1.00	5.46	97.00 ± 54.30	92.61	4.01 ± 2.60	3.95
Wamberg L. *et al.*^22^	7000	D group^b^	5.3 ± 0.4	5.4 ± 0.6	72.9 (50.0–89.5)^h^	60.0 (53.1-79.1)^h^	3.1 (2.1–3.5)^h^	2.4 (2.0–3.3)^h^
		Control group	5.5 ± 0.5	5.3 ± 0.6	82.2 (42.3–107.3)^h^	72.9 (52.0–92.1)^h^	3.2 (1.6–4.5)^h^	2.8 (2.3–3.7)^h^
Nagpal J. *et al.*^30^	12000	D group^b^	N/A	N/A	N/A	N/A	2.58 ± 16.99^g^	
		Control group					2.61 ± 18.29^g^	

^a^Mean and range.

^b^Group receiving vitamin D supplementation.

^c^Vitamin D was administered once a week.

^d^Additional calcium supplementation (500-1200 mg/d).

^e^Values are represented as mean ± SE.

^f^Log-transformed variables are presented as geometric means (95% CIs).

^g^Changes where p = 0.995.

^h^Median (25%; 75%). * - The statistically significant changes (p < 0.05), N/A - not available.
