# Supplementary material for: The effect of vitamin D supplementation on insulin and glucose metabolism in overweight and obese individuals: systematic review with meta-analysis
Source: Sci Rep. 2015 Nov 6;5:16142. doi: 10.1038/srep16142 (PMC4635423; doi:10.1038/srep16142)
Supplement: Supplementary File [file srep16142-s1.pdf]

Supplemental file

The effect of vitamin D supplementation on insulin and glucose metabolism in overweight and obese individuals: systematic review with meta-analysis

Małgorzata Jamka[1], Małgorzata Woźniewicz[1], Jan Jeszka[1], Marcin Mardas[1,2], Paweł Bogdański[3] & Marta Stelmach-Mardas[4*]

[1]Department of Human Nutrition and Hygiene, Poznan University of Life Sciences,

31 Wojska Polskiego Str., 60-624 Poznan, Poland

[2]Department of Oncology, Poznan University of Medical Sciences,

82/84 Szamarzewskiego Str., 60-569 Poznan, Poland

[3]Department of Education and Obesity Treatment and Metabolic Disorders, Poznan University of Medical Sciences, Poland

84 Szamarzewskiego Str., 60-596 Poznan, Poland

[4]German Institute of Human Nutrition Potsdam-Rehbruecke,

114-116 Arthur-Scheunert-Allee, 14558 Nuthetal, Germany

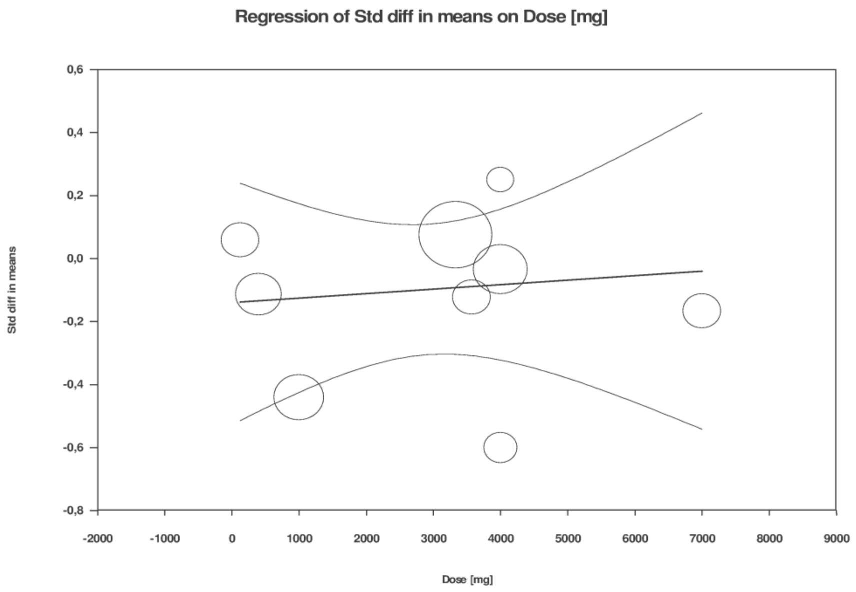

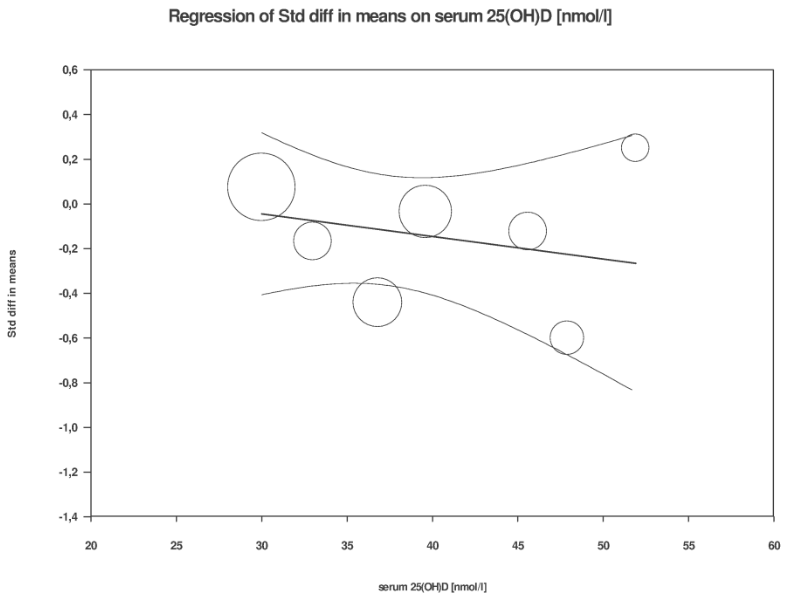

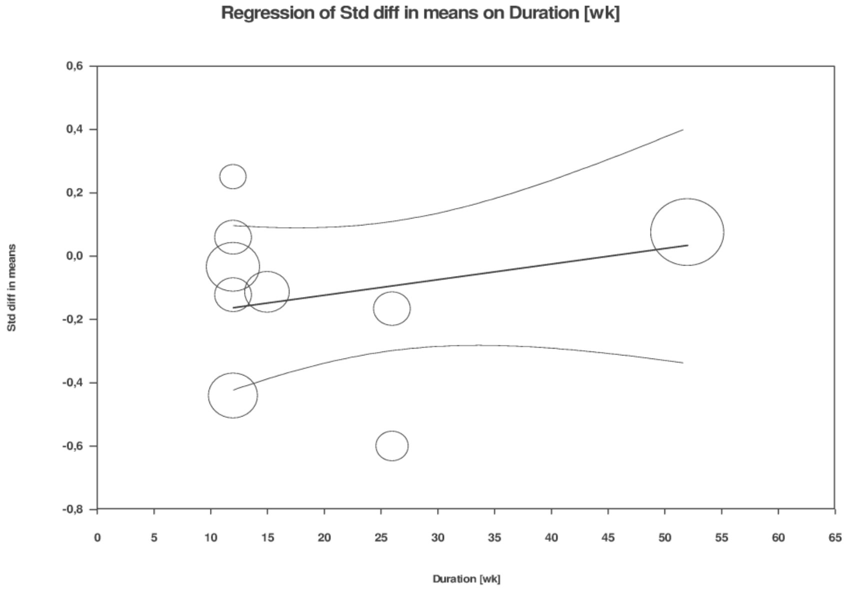

Figure 6. Estimated std. differences in means of impaired glucose events in selected randomized trials of plasma fasting glucose reduction according to A. the dose of vitamin D supplementation B. time of vitamin D supplementation and C. baseline of 25(OH)D concentration used in each trial.

*The circle corresponding to each study has area inversely proportional to the variance. The superimposed line was obtained by regression using the REML estimate of the residual heterogeneity variance. There was no significant association between **A**. supplemented dose of vitamin D (β= 0.000; SE: 0.000, 95%CI:-0.0001-0.0001; z= 0.31; p= 0.7562) **B**. time of vitamin D supplementation (β= 0.0049; SE: 0.0048, 95%CI:-0.0045- 0.0144; z=1.02; p= 0.3081) **C**. baseline of 25(OH)D concentration (β= -0.0101; SE: 0.0147, 95%CI:-0.0389 – 0.0187; z=- 0.69; p= 0.4915) and glucose level

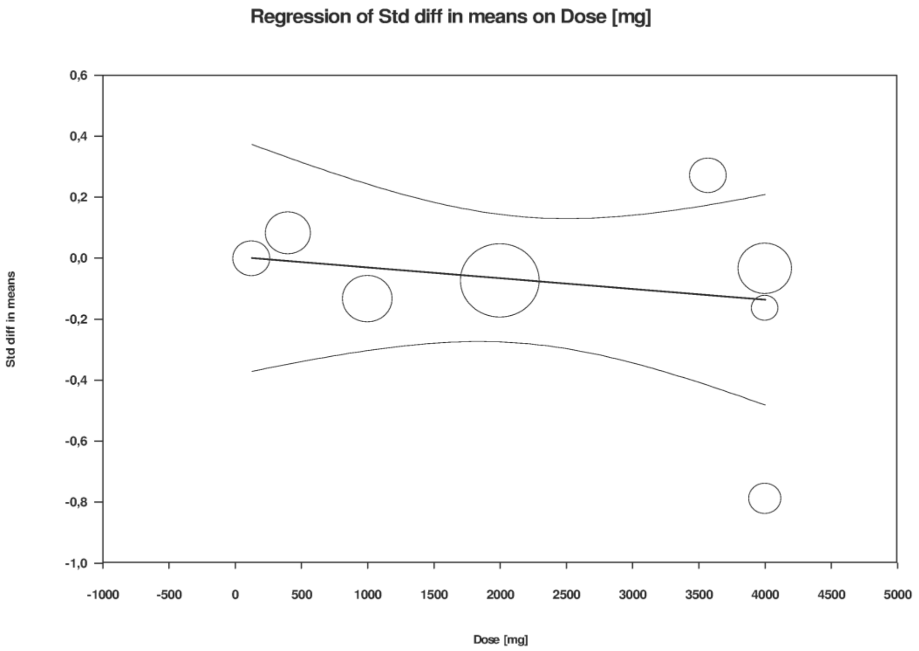

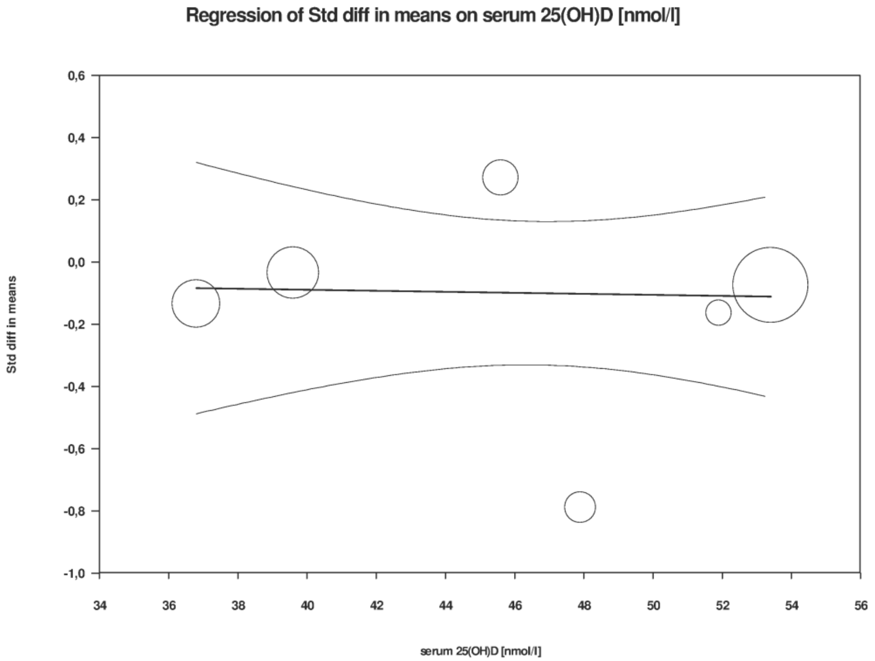

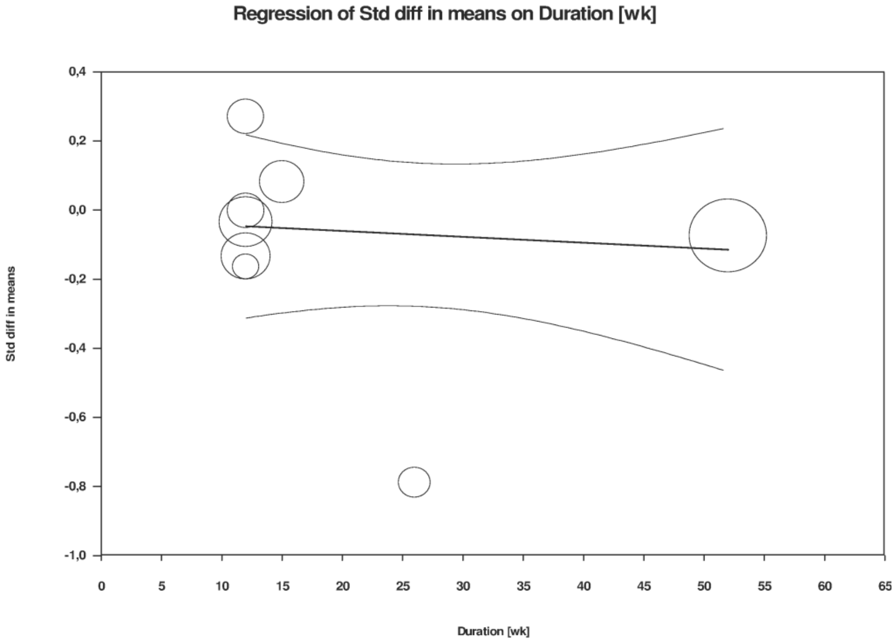

Figure 7. Estimated std. differences in means of higher insulin levels events in selected randomized trials of plasma insulin reduction according to A. the dose of vitamin D supplementation B. time of vitamin D supplementation and C. baseline of 25(OH)D concentration used in each trial.

*The circle corresponding to each study has area inversely proportional to the variance. The superimposed line was obtained by regression using the REML estimate of the residual heterogeneity variance. There was no significant association between **A**. supplemented dose of vitamin D (β= -0.000; SE: 0.0001, 95%CI:-0.0002-0.0001; z= -0.58; p= 0.5644) **B**. time of vitamin D supplementation (β= -0.0017; SE: 0.0046, 95%CI:-0.0108- 0.0074; z=- 0.36; p= 0.7175) **C**. baseline of 25(OH)D concentration (β= -0.0017; SE: 0.0138, 95%CI:-0.0286 – 0.0253; z=- 0.12; p= 0.9039) and insulin level.

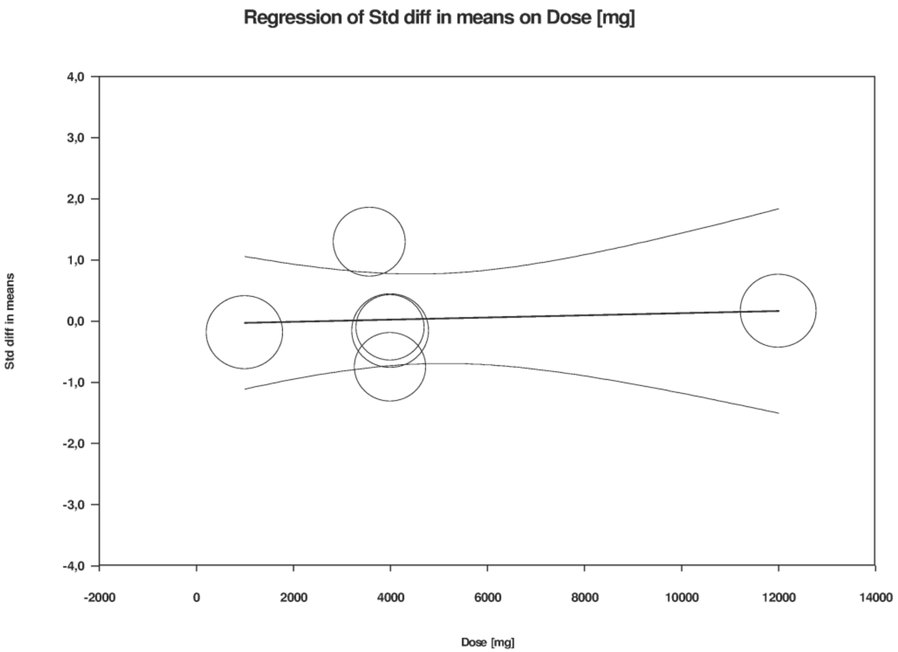

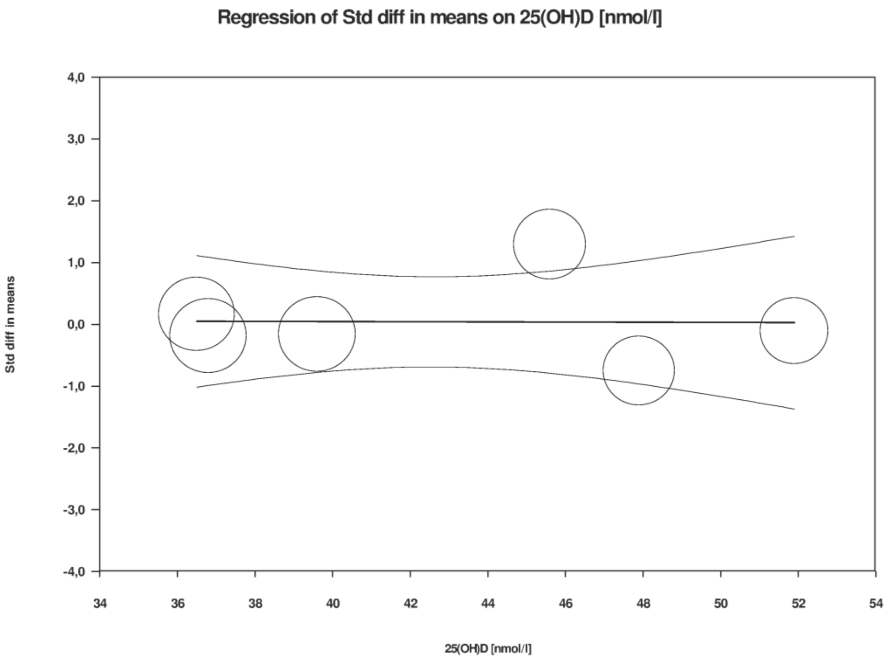

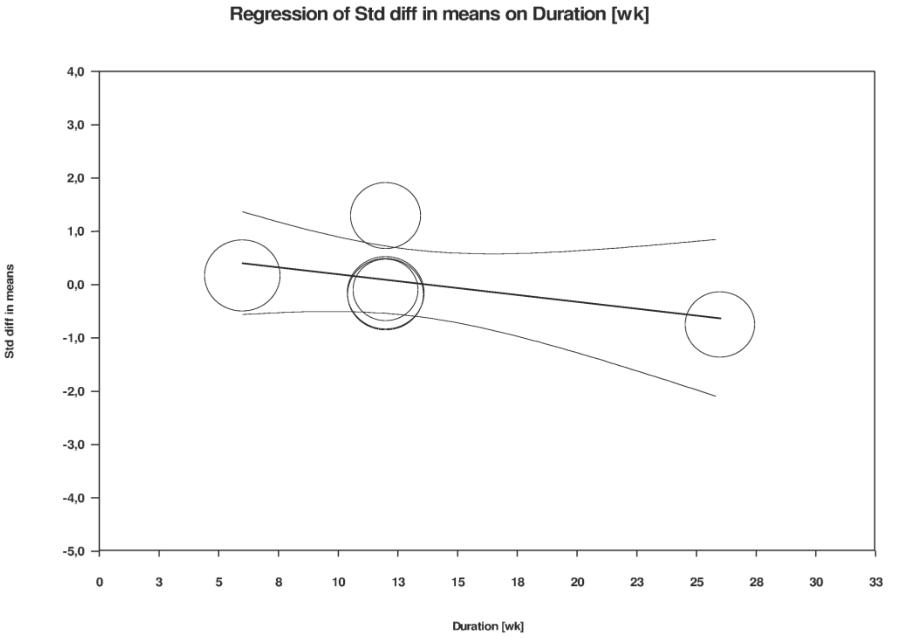

Figure 8. Estimated std. differences in means of insulin resistance events in selected randomized trials of HOMA-IR index reduction according to A. the dose of vitamin D supplementation B. time of vitamin D supplementation and C. baseline of 25(OH)D concentration used in each trial.

*The circle corresponding to each study has area inversely proportional to the variance. The superimposed line was obtained by regression using the REML estimate of the residual heterogeneity variance. There was no significant association between **A.** supplemented dose of vitamin D (β= 0.000; SE:0.0001, 95%CI:-0.0001-0.0002; z= 0.20; p= 0.8386) **B**. time of vitamin D supplementation (β= -0.0518; SE: 0.0427, 95%CI:-0.135- 0.032; z=- 1.21; p= 0.2253) **C**. baseline of 25(OH)D concentration. β= -0.0013; SE: 0.0522, 95%CI:-0.103 – 0.101; z=- 0.33; p= 0.9794) and the HOAM-IR index
